# The neural basis of weight control and obesity

**DOI:** 10.1038/s12276-022-00759-3

**Published:** 2022-04-26

**Authors:** Min-Seon Kim

**Affiliations:** 1 Division of Endocrinology and Metabolism, Asan Medical Center, University of Ulsan College of Medicine, Seoul, Korea; 2 Appetite Regulation Laboratory, Asan Institute for Life Sciences, University of Ulsan College of Medicine, Seoul, Korea

**Keywords:** Endocrine system and metabolic diseases, Feeding behaviour

According to the World Health Organization’s report, approximately 13% of the world’s adult population was obese, and 39% was overweight in 2016. The worldwide prevalence of obesity nearly tripled between 1975 and 2016^1^. Overconsumption of calorie-dense foods and a sedentary lifestyle are two main drivers of the global obesity epidemic. Despite obvious causes of obesity, our efforts to combat obesity have been unsuccessful. To effectively treat obesity, we need to understand how our body maintains a normal weight and what pathological processes disrupt weight control mechanisms.

The most important determinant of body weight is the amount of caloric intake over weeks and months. Indeed, human subjects with morbid obesity commonly manifest binge eating. The purpose of eating is to live (for energy) or for pleasure. The former is called homeostatic feeding, and the latter is called hedonic feeding. The hypothalamus has long been regarded as a key regulating center of feeding behavior. For homeostatic feeding, the neurons in the mediobasal hypothalamus (MBH) sense the energy state of the body through changes in hormones (leptin, insulin, ghrelin, etc.) and nutrients (glucose, fatty acids, amino acids, etc.) in the bloodstream^2^. This metabolic information is processed by MBH neurons and transmitted to several intra- and extrahypothalamic regions that control feeding behavior. Eventually, feeding behaviors are promoted or suppressed depending on the energy level of the body. Information about energy intake arising from the gastrointestinal (GI) tract is also delivered to the hypothalamus via gut-innervated neurons and hindbrain pathways^3^. Hedonic feeding is triggered by the sensory pleasure caused by foods and the related contextual memories. It involves the lateral hypothalamus and dopaminergic reward system. Advances in neuroscience technology have enabled the identification of previously unsuspected neurons and neural circuits that engage homeostatic and hedonic feeding. Recent discoveries on feeding-regulatory brain subregions, types of neurons and their circuits are illustrated in a paper of this special series (Ahn et al.: 10.1038/s12276-022-00758-4).

Another critical factor determining body weight is energy expenditure. Energy is expended for basal metabolism, physical activity, and adaptive thermogenesis. The basal metabolic rate (BMR) is the minimal amount of calories we burn to maintain basic life-sustaining functions, and it makes up the largest proportion (approximately 60–70%) of total energy expenditure. Skeletal muscle is the largest organ and a major contributor to BMR. Thus, BMR increases in proportion to lean body mass. The energy expenditure used for physical activity varies substantially from day to day and between individuals. The rest of the energy expenditure is used for adaptive thermogenesis, i.e., cold-induced thermogenesis and diet-induced thermogenesis. The primary organ for nonshivering thermogenesis is brown adipose tissue (BAT). In humans, functional BAT has been found in several fat depots, and the activity of BAT negatively correlates with body mass index (BMI)^4^. Similar to feeding regulation, the hypothalamus is a central controller of three components of energy expenditure. Neuronal mechanisms mediating the hypothalamic regulation of energy expenditure are outlined in a paper of this special series (Tran et al.: 10.1038/s12276-022-00741-z).

The central nervous system (CNS) communicates with peripheral metabolic organs to maintain energy homeostasis. These communications mainly occur through the autonomic nervous system (ANS). The ANS controls both food intake and energy expenditure. The parasympathetic vagus nerve relays information on food ingestion to the CNS and contributes to satiety formation by controlling gastric motility and emptying^5^. However, the sympathetic nervous system seems to play a dominant role in regulating energy expenditure, as adipose tissues are only innervated by sympathetic nerves^6^. In addition to energy metabolism, the brain modulates glucose metabolism in multiple peripheral organs by means of the ANS. A review article in this special series (Hyun et al.: 10.1038/s12276-021-00705-9) highlights the ANS regulation of energy and glucose metabolism.

The gut-brain axis has recently drawn much attention in obesity research due to the significant success of gut-targeting anti-obesity treatments, such as bariatric operations and glucagon-like peptide-1 (GLP-1) agonists. The gut informs the CNS about meal size and composition through the secretion of hormones (GLP-1, cholecystokinin, etc.) and mechanical stretch. The GI tract contains the enteric nervous system (ENS), which regulates gut motility, metabolism, and immune functions. CNS neurons modulate ENS neuronal activity and functions via the ANS and spinal nerves^7^. Approximately 10 trillion microbes reside in the human GI tract. The symbiotic gut microbes significantly impact the host’s energy and glucose metabolism by regulating gut functions and releasing small metabolites into the bloodstream^8^. The gut microbiota plays a crucial role in helping us extract nutrients and energy from foods. Conversely, obesity alters the gut microbial composition and diversity, resulting in further deterioration of energy and glucose metabolism. The current understanding of the roles of the gut-brain axis in energy and glucose regulation is described in a paper in this special series (Wachsmuth et al.: 10.1038/s12276-021-00677-w).

Overconsumption of a high-fat diet (HFD), especially a diet high in saturated fatty acids (SFAs), is closely associated with human obesity. Persistent exposure to a HFD induces inflammation and innate immune responses in the MBH. Hypothalamic inflammation impairs the ability of hypothalamic neurons to sense the peripheral energy state and to modulate feeding and energy expenditure. This eventually causes neurodegeneration and the death of MBH neurons^9^. Thus, neuroinflammatory responses to SFAs are regarded as a key pathological mechanism of hypothalamic dysfunction during obesity progression. CNS-resident immune cells and glia play important roles in the process of hypothalamic inflammation. A review article in this special series (Folick et al.: 10.1038/s12276-021-00666-z) illustrates how various metabolic factors modulate hypothalamic innate immune responses in obesity.

Maternal obesity and metabolic disorders can be transmitted to children^10^. Maternal overnutrition and undernutrition during pregnancy and lactation increase the risks of obesity and metabolic disorders in children later in life. In the early postnatal period, leptin is secreted from adipocytes and acts as a metabolic cue for neural circuit organization in the moue hypothalamus^11^. Maternal HFD consumption disrupts leptin signaling in the developing hypothalamus and disrupts hypothalamic circuit organization^11^. This developmental defect in hypothalamic circuits may promote obesity and abnormal glucose metabolism in adulthood. The mechanisms of developmental programming of hypothalamic melanocortin circuits, a critical regulator of body weight, are discussed in an article in the special series (S. G. Bouret: 10.1038/s12276-021-00625-8).

In recent decades, our knowledge of the neural mechanisms of weight control has constantly expanded thanks to advances in neuroscience and genetic technology. A better understanding of this critical body function will lead us to develop successful pharmacological therapies to combat obesity.
